# Phloem-Feeding Herbivores Affect Floral Development and Reproduction in the Etruscan Honeysuckle (*Lonicera etrusca* Santi)

**DOI:** 10.3390/plants10040815

**Published:** 2021-04-20

**Authors:** Sandra V. Rojas-Nossa, José María Sánchez, Luis Navarro

**Affiliations:** 1 Department of Ecology and Animal Biology, Campus Lagoas-Marcosende, University of Vigo, 36310 Vigo, Spain; 2 Department of Plant Biology and Soil Sciences, Campus Lagoas-Marcosende, University of Vigo, 36310 Vigo, Spain; jmsbot@uvigo.es (J.M.S.); lnavarro@uvigo.es (L.N.)

**Keywords:** aphid, flowering, *Hyadaphis passerinii*, nectar, pollination, reproductive success, stigma

## Abstract

Floral development depends on multifactor processes related to genetic, physiological, and ecological pathways. Plants respond to herbivores by activating mechanisms aimed at tolerating, compensating, or avoiding loss of biomass and nutrients, and thereby survive in a complex landscape of interactions. Thus, plants need to overcome trade-offs between development, growth, and reproduction vs. the initiation of anti-herbivore defences. This study aims to assess the frequency of phloem-feeding herbivores in wild populations of the Etruscan honeysuckle (*Lonicera etrusca* Santi) and study their effects on floral development and reproduction. The incidence of herbivory by the honeysuckle aphid (*Hyadaphis passerinii* del Guercio) was assessed in three wild populations of the Iberian Peninsula. The effect of herbivory on floral morphology, micromorphology of stigmas and pollen, floral rewards, pollination, and fruit and seed set were studied. The herbivory by aphids reduces the size of flowers and pollen. Additionally, it stops nectar synthesis and causes malformation in pollen and microstructures of stigmas, affecting pollination. As a consequence, fruit set and seed weight are reduced. This work provides evidence of the changes induced by phloem-feeding herbivores in floral development and functioning that affect the ecological processes necessary to maintain the reproductive success of plants.

## 1. Introduction

A diversity of organisms can act as selective agents for plants by directly and indirectly influencing their reproductive success [1]. Such interactions are rarely isolated and may result in changes in plant population dynamics and evolutionary traits, potentially impacting other participants within interaction networks [2]. Since sexual reproduction of most angiosperms relies on animals obtaining a reward for transporting pollen among flowers, plant-pollinator interactions are cornerstones for terrestrial ecosystem functioning [3,4]. However, pollination interactions are permanently under the effects of a diversity of factors.

Herbivores have contrasting consequences for plants at different ecological levels. The direction and magnitude of the effects for the host are highly dynamic. These depend on the identity of the interacting species and their ability to respond to diverse environmental pressures. For example, foliar and floral herbivores are known because they cause a decrease in the production or quality of pollen. They can also destroy structures directly affecting plant reproduction and, thus, the population structure [5,6]. Herbivores also indirectly modify plant fitness by inducing changes in plant-pollinator interactions via reduction in the attraction and rewards for pollinators [7,8,9]. Not all interactions with herbivores have negative consequences for the plant. Several plant species are able to develop mechanisms to tolerate, compensate, or avoid the effects of this interaction [10,11], and in particular circumstances, increase plant reproductive success despite herbivory [12,13].

Aphids (Hemiptera, Aphioidea) are the largest group of phloem-feeding herbivores [14]. Usually, they are detrimental for host plants because they are vectors of infections, induce the production of leaf galls, or cause defoliation [15,16]. Aphids are common herbivores in many plant species including several crops [17]. However, knowledge about plant resistance mechanisms, responses, and especially the effects for the pollination of wild plant species is still emerging [18,19], but see [20,21,22].

*Lonicera etrusca* is a common perennial shrub that requires pollinators for reproduction [23]. The tubular flowers produce a large amount of nectar and are pollinated by legitimate visitors and nectar robbers such as hawkmoths, bumblebees, and carpenter bees [24,25]. Previous observations suggest that herbivory by aphids affects the production of fruits and seeds [24]. Nevertheless, the effects of these herbivores for the reproduction of the plant and the pathways through which aphids affect pollination have not been examined so far. The aims of this work are: (i) to assess the frequency of herbivory by aphids in *L. etrusca*; (ii) to study the consequences of herbivory for floral development; and (iii) to assess the effects of these herbivores on pollination and fitness.

## 2. Results

### 2.1. Frequency of Herbivory by Aphids

**Figure 1:**
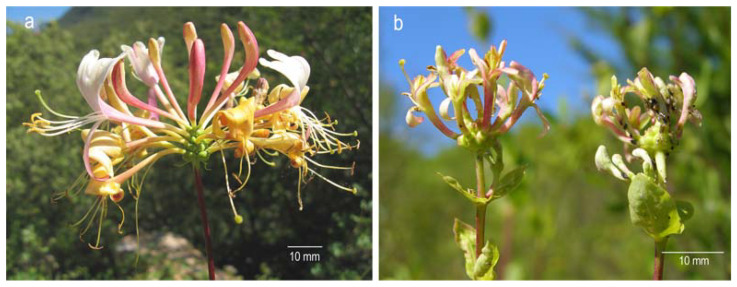
Flowers of *Lonicera etrusca*: (**a**) Healthy flowers. (**b**) Damaged flowers as a consequence of phloem-feeding by *Hyadaphis passerinii*. In this image, the inflorescence on the left shows flowers damaged by aphids that are no longer present, and the one on the right is damaged with aphids present.

Aphids consumed phloem from stems and pedicels of young leaves. Occasionally some were also present feeding in damaged buds and flowers during the blooming season, but not in healthy flowers (Figure 1). The overall proportion of flowers damaged by herbivory was 38.7% (*n* = 2703). Per population, percentages were 29.9% (*n* = 2846) in ‘La Barosa’ 30.1% (*n* = 1617) in ‘Cobas A,’ and 52.2% (*n* = 2842) in ‘Cobas B’. If the data from all three populations were pooled, the number of damaged flowers per plant was 14%.

### 2.2. Effects of Herbivory by Aphids on Floral Traits

**Figure 2:**
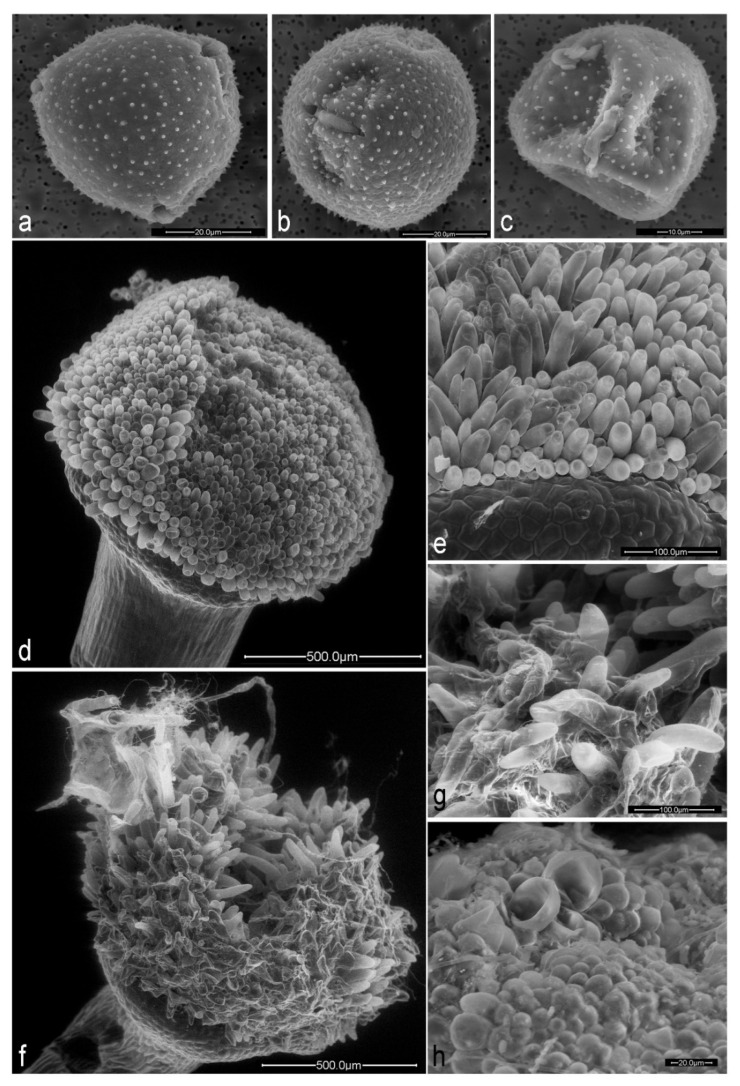
Morphology of pollen grains and stigmas of *Lonicera etrusca* with SEM. (**a**) Healthy pollen grain in polar view and (**b**) healthy pollen in semi-equatorial view; (**c**) typical pollen grain of a damaged flower; (**d**) stigmatic surface of a healthy flower; (**e**) detail of stigmatic papillae of a healthy flower; (**f**) stigmatic surface of a typical damaged flower; (**g**) and (**h**) detailed view of damaged stigmatic papillae.

Herbivory by *H. passerinii* on young stems and in floral buds produced detrimental changes in floral development (Figure 1), including both floral morphology and production of rewards for pollinators. Flowers affected by aphid herbivory (‘damaged flowers’ hereafter) were significantly smaller than non-affected flowers (‘healthy flowers’ hereafter) in all morphological variables assessed (Table 1). The shape, size, and quantity of gametes (damaging ovules and pollen) were also significantly affected by aphids (Figure 2a–c). The stigmatic papillae of damaged flowers are malformed, thus reducing the area for pollen reception (Figure 2d–h). Damaged flowers produced no nectar (Table 1).

### 2.3. Effects of Herbivory on Plant Reproduction

**Figure 3:**
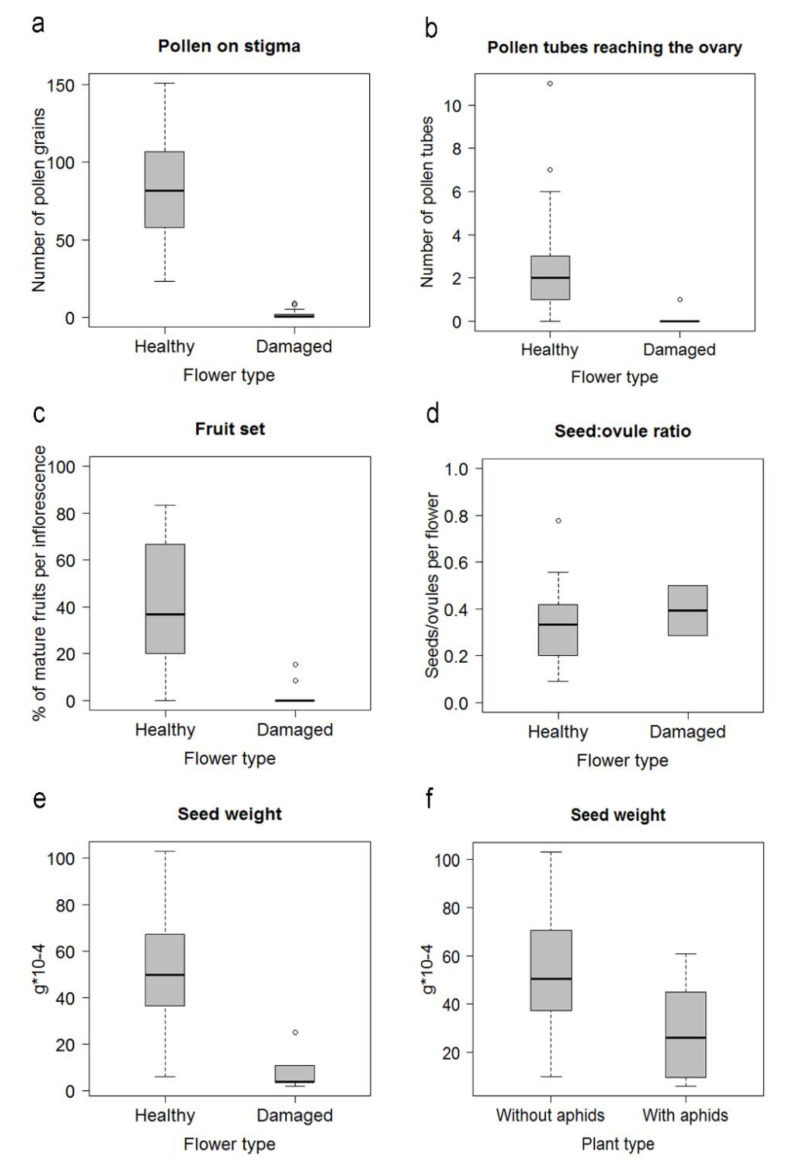
Consequences of herbivory by the phloem-feeding herbivore *Hyadaphis passerinii* for different parts of the pollination and post-pollination processes. (**a**) Arrival of pollen to stigmas; (**b**) pollen tubes growing down to the ovary; (**c**) fruit set; (**d**) number of seeds/number of ovules per ovary (Seed:ovule ratio); (**e**) weight of the seeds from healthy and damaged flowers; (**f**) weight of the seeds from healthy flowers from plants without aphids, and plants highly infested by aphids (with aphids). Box plots represent medians (horizontal lines), quartiles (boxes), 0.25−1.5 IQR and 0.75 + IQR (whiskers), and outliers (open dots).

**Figure 4:**
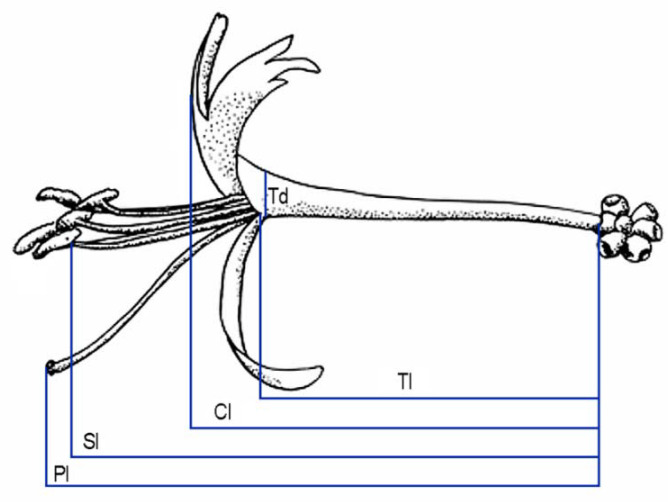
Floral traits characterised to assess the effects of herbivory by aphids on floral development of *Lonicera etrusca*: pistil length (Pl), stamen length (Sl), corolla length (Cl), tube length (Tl), and tube diameter (Td).

Phloem-feeding by aphids negatively affected the reproduction of *L. etrusca*. The quantity of pollen deposited on stigmas of damaged flowers was significantly lower than healthy flowers (*F*_1, 59_ = 157.99, *p* < 0.001. Figure 3a). Furthermore, the few pollen grains arriving at the stigma of damaged flowers seldom produced pollen tubes (*F*_2, 59_ = 13.84, *p* < 0.001. Figure 3b) in comparison with healthy flowers (Figure 4). In addition, aphids affected the development of the whole inflorescence. Consequently, the percentage of ripe fruits per inflorescences was lower in those branches infested by aphids (χ^2^ = 41.21, df = 1; *p* < 0.001. Figure 3c). Although the fruits produced from damaged and healthy flowers had a similar number of seeds (Figure 3d), the seeds from damaged flowers were noticeably lighter than those from healthy flowers (Figure 3e). The effects were similarly noticeable at the plant level, as healthy flowers on plants strongly affected by aphids produced significantly lighter seeds than those produced in healthy flowers on unaffected plants (*t* = 2.89, df = 85, *p* < 0.005, and Figure 3f).

## 3. Discussion

### 3.1. Effects of Herbivory on Floral Development

The water and nutrients in phloem sap that are exploited by the aphids are necessary for plant development [26]. Those are limited resources needed for different plant functions [27], so a number of trade-offs arise between development, growth, and reproduction vs. the induction of anti-herbivore defences in plants [28]. Aphids and other herbivores are known for causing a decrease in number or size of flowers, and number or size of pollen grains and ovules [8,9,20,29].

In some instances, aphids can suppress the budding of some shoots altogether [19]. Two main nonexclusive hypotheses explaining flowering decrease as a consequence of aphids are the biochemical interaction with the physiology of the plant, and the sequestering of photosynthates from the flowering process [19]. The first hypothesis would explain the absence of budding in the first place, but the latter is a more likely explanation for *Lonicera* since buds are produced but cannot develop into whole healthy flowers.

Moreover, the low weight of seeds produced in fruits from healthy flowers of highly infested plants seem to suggest that herbivores modify the resource budget at the individual level with effects on reproductive traits. Nectar absence in damaged flowers is due to the malformation or damage of the nectaries, thus physically preventing nectar synthesis. Considering that aphids are important pests of crops and wild plants, further research is needed to understand their effects on physiological pathways and ecological processes.

The high levels of infestation per plant are related to the high rates of aphid population growth [30]. These herbivores decipher chemical compounds to determine environmental conditions, particularly related to mate and host location, even being able to detect the physiological condition of the plants [31]. Other traits involved in a plant’s capacity to respond to herbivory, beyond the scope of this work, may also be related to the observed differences in infestation levels [32].

### 3.2. Effects of Herbivory on Plant Reproduction

Other studies reported a decrease in nectar production caused by herbivores [9]. Nevertheless, the total disruption of nectar synthesis recorded for *L. etrusca* in this study is remarkable. This fact, together with changes in the floral display, are the most likely causes for the reduction in pollination quality observed. In the studied populations, a damaged flower is significantly less likely to be visited by nectar robbers, easily noted by the absence of the holes made by them [33]. Since bumblebees and carpenter bees behaving as nectar robbers are also effective pollinators of *L. etrusca* [25], this indicates that aphids are indirectly inducing changes in the foraging behaviour of pollinators. A reduction in pollinator visit frequencies caused by floral and foliar herbivory was reported for several plant species and linked to a decrease of floral rewards (pollen and nectar), changes in the floral display, and production of secondary compounds [7,8,9]. The activation of anti-herbivore defences, and the trade-offs among plant defence, growing, and reproduction [1,34] are some of the mechanisms worth exploration in future studies.

Aphids commonly feed on young stems and pedicels of leaves before blooming, resulting in the development of damaged flowers. During the blooming season, we observed these insects also behaving as florivores, feeding on floral tissues. This was only observed in damaged flowers and we did not find aphids in healthy flowers. The consumption of phloem by aphids in inflorescences might increase detrimental effects for the whole plant, through the continual depletion of nutritional resources. An interesting evolutionary explanation of the interaction between plants and aphids has been hypothesised by Watanabe et al. [19]; since aphids are detrimental to plant fitness, plants defend themselves by modifying the biochemical composition of the phloem, thereby killing most aphids during the blooming season, establishing an evolutionary arms race between them. There are commonalities between *Lonicera* and *Artemisia montana* [19], as the detrimental effects for the plant and the elimination of most aphids at the end of the flowering period suggest a similar arms race could be at work here. Additional data on the biochemical relationships between aphids and plants could help to ascertain that hypothesis. The presence of these insects in the plants also attracts other animals with potential effects for the host, such as ants that feed on aphid honeydew or spiders that predate aphids (S. Rojas-Nossa Pers. Obs.). Ants are known because they actively defend aphids against possible predators and are able to induce changes in pollinator behaviour modifying the reproductive success of the host plants [35]. Further research to understand the physiological and ecological effects of phloem-feeding herbivores is needed.

Pollen tubes reaching the ovary have been considered a measure of fitness for this plant species [25], although some late-acting effects can result in different fitness values when other indicators such as fruit or seed production are considered. In this study, we have considered all three of them, finding that the pollination is highly affected, but some damaged flowers are able to produce fruits and seeds. The flowers of this plant are visited by a variety of insects that feed on pollen, nectar, or both, and behave in different manners as well [24]. One possible explanation for the successful fruit setting of some damaged flowers is that the pollinator foraged for pollen and not only for nectar. Additionally, some floral visitors have different capacities to assess nectar content before visiting the flower [36,37], but some others are unable to do it. Thus, the latter are obliged to visit the flower regardless of the reward contained. In these cases, nectarless flowers could still be visited and, thus, pollinated. Actually, diverse plant species have a broad variability in nectar production, and it has been hypothesised that this uncertainty for pollinators is an ecological and evolutionary mechanism of plants to maintain pollination and increasing outcrossing while reducing costs of nectar production [38]. On the top of that, a recent work provides evidence of pollination of the species by nectar robbers that crawl on the inflorescences and flowers to perforate the corolla and extract nectar [25]. Pollen carryover between flowers is facilitated by the exerted reproductive structures and big size of nectar robbers. During the field work we observed signs of nectar robbing on damaged flowers, although the frequency is very low in comparison with robbing on healthy flowers (1.1% *n* = 358 vs. 58.8% *n* = 505, respectively. S. Rojas-Nossa, unpublished data). Additionally, the analysis of the signs left by nectar robbers on the corollas revealed that the visits by robbers in damaged flowers were performed by coleopterans, which make a particular long tear easily to differentiate from the holes or slits made by other robbers such as bumblebees or carpenter bees [33]. Probably, coleopterans are unable to assess nectar content before visiting a flower. Moreover, this insect group consumes both pollen and nectar, so they can still obtain a reward in damaged flowers despite the absence of nectar or the malformation of pollen grains. If this is the case, coleopterans and other pollen foragers are likely candidates to be responsible for the pollination of damaged flowers; this is something that deserves further attention.

## 4. Materials and Methods

### 4.1. Study Sites

The research was conducted at three populations in northwest Spain: Cobas A (42°28’19’’ N, 6°50’17’’ W 567 m asl) and Cobas B (438 m asl 42°28’15’’ N, 6°49’26’’ W) are located in the Natural Park Serra da Enciña da Lastra, municipality of Rubiá, province of Orense, and La Barosa (590 m asl 42°29’50’’ N, 6°48’52’’ W) in the municipality of Carucedo, province of León. The region has a Mediterranean climate, and the landscape is a mosaic of cultivated lands and native vegetation, such as shrub communities and holm oak woodland (predominantly *Arbutus unedo*, *Quercus ilex* and *Q. suber*) [39].

### 4.2. Study System

*Lonicera etrusca* (Caprifoliaceae) is a climbing shrub of the Mediterranean Basin. The flowers have a long sympetalous corolla, which open at dusk and last three days [24]. The evidence reveals that the plant does not produce fruits when stigmas receive pollen from the same flower; thus, visits by pollinators are necessary to achieve sexual reproduction [23,25]. Its berries are an important resource for frugivorous animals [24].

The honeysuckle aphid *Hyadaphis passerinii* (Aphidinae: Macrosiphini) is widespread in Europe, predominantly in the Mediterranean Region [40]. It is a herbivore of some species of the *Lonicera* genus, particularly *L. caprifolium* and *L. periclymenum*, forming colonies in early spring. When winged individuals are produced during the summer, they often migrate to plants of the genera *Daucus*, *Conium,* and *Pastinaca* [40]. Herbivory by *H. passerine* causes damages on shoot growth and flower development [41].

### 4.3. Frequency of Herbivory by Aphids

Three transects of 300 m length and 5 m width were established, with one transect per study site. We observed that flowers produced on young stems that suffer herbivory by aphids were malformed. These flowers were named as ‘damaged’ to differentiate them from flowers developing on stems without herbivory that were named ‘healthy’. In spring, we counted the total number of flowers within the transects and inspected a random sample of flowers, with a maximum of three flowers per individual (914 flowers in Cobas A, 1192 in Cobas B, and 597 in La Barosa). The inspected flowers were assigned to one of the two groups (healthy or damaged) to assess the frequency of herbivory by aphids in the studied populations.

### 4.4. Effect of Herbivory on Floral Development

To study changes in floral development caused by herbivory, we marked floral buds of the two groups. To study changes in floral morphology, 146 fresh flowers (83 healthy and 63 damaged) from 40 individual plants sampled after anthesis in three populations (14 plants in Cobas A, 14 in Cobas B, and 12 in La Barosa) were measured with a digital calliper (0.01 mm precision). The floral traits characterised were total corolla length, tube length, tube diameter, longest stamen length, and pistil length (Figure 4).

To assess the effect on the number of ovules per flower, 40 flowers (20 damaged and 20 healthy) were randomly collected from 40 plants and preserved in vials with 70% ethanol. In the laboratory, the ovaries were dissected, and the number of ovules were counted under a stereoscopic microscope. To measure the ovule size, a random sample of five ovules per flower (*n* = 200 ovules from 40 plants) was placed on a microscope slide with a micrometre scale and photographed; finally, we measured length and width of each ovule with the ImageJ software (version 1.46r for Windows. NIH, Bethesda, MD, USA).

To quantify the number of pollen grains per anther, 40 floral buds were collected (one per plant, 12–15 plants per population in three populations) and preserved in vials with 70% ethanol. At the laboratory, one anther was randomly chosen per flower. Then it was placed on a microscope slide with a drop of isotonic solution (ISOTON II) and all pollen grains were manually removed with dissecting needles under a stereoscopic microscope. Grains were counted with a Multisizer Coulter Counter, Beckman Coulter (see [42] for a thorough description). To characterize pollen size, one anther per flower from 20 different plants was dissected under a stereoscopic microscope (10 anthers of each group of flowers) and placed on a microscope slide with a glycerine drop. Measurements of equatorial and polar axis of 10 pollen grains per anther (*n* = 100) were made with a reticle in the light microscope. To examine the microscopic structure of pollen and stigmas, a sample of 30 healthy and 30 damaged flowers was randomly taken and preserved in 70% ethanol. One flower of each type was taken from each plant (*n* = 30 individual plants) to control for variation among maternal plants. At the laboratory, pollen and stigmas were separated and dehydrated with increasing concentrations of aqueous ethanol solutions (70–100%). Afterwards ethanol was replaced with amiloacetate (successive amiloacetate-ethanol solutions 1:3, 2:2, 3:1). Then, samples were treated with a critical point drier and mounted on metallic stubs. Pollen samples were coated with a gold/palladium film under high vacuum in a sputtering chamber. Samples were then observed and photographed with an environmental scanning electron microscope (SEM) in low vacuum mode, operating at 15 kV. To compare the volume and sugar concentration of nectar between healthy and damaged flowers, 110 floral buds (83 healthy and 27 damaged from 90 individuals) were bagged with mosquito net bags. The nectar accumulated after 24 h was extracted with 1 µL capillary micropipettes. Sugar concentration was estimated with a portable refractometer (Fisher Scientific TM, 0–50%, Waltham, MA, USA).

### 4.5. Effect of Herbivory on Plant Reproduction

**Figure 5:**
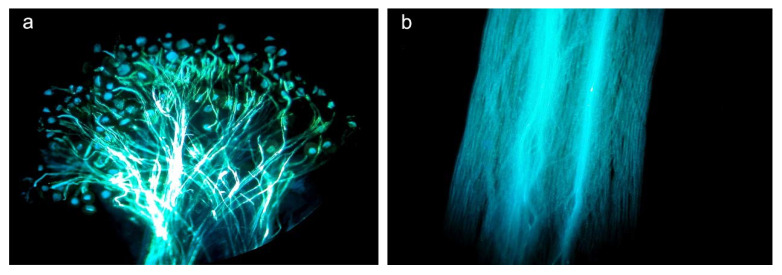
Pollen tube growth in healthy flowers. (**a**) Pollen germinating at the stigmatic surface. (**b**) Pollen tubes reaching lower part of the style.

To assess the effect of aphids on the number of pollen grains deposited on stigma and pollen tubes reaching the ovary, one flower of each type (healthy and damaged) was taken from each of 60 plants (20 plants per population) on the third day after anthesis. Third day flowers were visually identified by their yellowish perianth and dehiscent anthers. To assess pollen tubes growth, we performed the procedure described by [25] (Figure 5). To estimate the effect of herbivory by aphids on fruit and seed set, 120 inflorescences (60 with healthy and 60 with damaged floral buds) were marked in 30 individuals (min. 1 and max. 6 inflorescences per individual). Two levels of infestation by aphids were characterised for each individual: plants without aphids and highly infested plants (more than 90% of the flowers damaged). To control for possible architectural effects on fruit production related to differential resource-availability [43], the position of the inflorescence on the stem was recorded as apical (*n* = 45), or lateral of first (*n* = 22), second (*n* = 23), or third order (*n* = 30). After fruit maturation, the infructescences were collected and preserved in ethanol 70%. At the laboratory, the number of ripe fruits and ovaries per infructescence were counted. One fruit per infructescence was dissected and the number of viable seeds and undeveloped ovules were counted. Finally, viable seeds were extracted from the fruit, cleaned, and dehydrated in an oven at 50 °C until constant weight. Seed weight was measured with an analytical balance (0.01 mg precision).

### 4.6. Data Analyses

To test for statistical differences in floral traits of healthy and damaged flowers, *t*-tests were performed. To compare the quantity of pollen grains received by the stigmas of healthy and damaged flowers, an analysis of variance (ANOVA) with 10,000 bootstraps was performed. The number of pollen tubes reaching the ovary was analysed with an analysis of covariance (ANCOVA) with 10,000 bootstrap iterations. The quantity of pollen on stigma was used as a covariate. We fitted Generalised Linear Mixed Models (GLMM) to analyse whether the damage by herbivores had an effect on the number of ripe fruits produced per inflorescence. The type of flower (healthy or damaged) was included as a fixed effect. The plant identity and the position of the inflorescence at the branch were used as random effects. We checked for overdispersion. The model was fitted with Poisson’s error structure and logit link function. The number of ovaries per inflorescence was included as an offset term (square root transformed) into the model. To test specifically for the effect of herbivory, we compared the full model with a null model lacking the type of flower but comprising all other terms in the full model. This comparison was made using a likelihood ratio test. Because the number of fruits produced by damaged flowers was very low, it was not possible to compare statistically the quantity or weight of seeds of healthy and damaged flowers. To compare the weight of seeds produced by healthy flowers from plants without aphids, and plants highly infested by aphids, a *t*-test was performed. For this analysis, the predictor variable was the plant level of infestation described above. We considered 0.05 as the level of significance. All analyses were performed with R software (version 2.15.1 for Windows [44]).

## 5. Conclusions

Herbivory by aphids has profound negative impacts on floral development and pollination of the host plant. As a result, male and female components of reproductive success are affected. This finding is all the more remarkable considering how prevalent damaged flowers were found to be in the studied populations.

## Figures and Tables

**Table 1:** Changes in floral traits caused by aphids.

Floral Trait ^1^	Flower Type Mean ± S. D. (Sample Size)		Paired Student’s *t*-Test
	**Healthy**	**Damaged**	
Nectar volume (µL)	4.7 ± 4.1 (83)	0 (27)	
Sugar concentration (◦Brix)	21.1 ± 6.3 (83)	-	
Total corolla length	30.9 ± 2.6 (83)	12.5 ± 2.3 (63)	*t* = −14.2, *p* = 0.001
Tube length	25.6 ± 1.8 (83)	9.2 ± 3.3 (63)	*t* = −14.3, *p* < 0.001
Tube diameter	2.4 ± 0.3 (83)	1.5 ± 0.3 (55)	*t* = −10.9, *p* < 0.001
Pistil length	42.7 ± 2.8 (83)	14.8 ± 3.7 (63)	*t* = −9.5, *p* < 0.001
Stamens length	39.4 ± 3.0 (83)	12.6 ± 3.0 (63)	*t* = −13.9, *p* < 0.001
Ovules/flower	11 ± 1.2 (20)	8.8 ± 2.3 (20)	*t* = 3.67, *p* = 0.001
Ovule length	1.3 ± 0.1 (100)	1.1 ± 0.1 (100)	*t* = 5.1, *p* < 0.001
Ovule width	0.8 ± 0.1 (100)	0.6 ± 0.1 (100)	*t* = 4.9, *p* < 0.001
Pollen grains per anther	2362 ± 366 (20)	1447.4 ± 757 (20)	*t* = −4.8, *p* < 0.001
Pollen equatorial axis (µm)	71.1 ± 3.5 (50)	59.4 ± 5.3 (50)	*t* = 9.7, *p* < 0.001
Pollen polar axis (µm)	63.2 ± 3.4 (39)	55.2 ± 5.2 (50)	*t* = 7.7, *p* < 0.001

^1^ Morphometric measurements are reported in mm.

## Data Availability

Data available from the Figshare Digital Repository: < 10.6084/m9.figshare.14222474>.
